# B Vitamins and Cognitive Performance in Older Adults: Review

**DOI:** 10.5402/2013/650983

**Published:** 2013-03-11

**Authors:** J. L. Reay, M. A. Smith, L. M. Riby

**Affiliations:** ^1^School of Social Sciences and Law, Teesside University, Middlesbrough, Tees Valley TS1 3BA, UK; ^2^Department of Psychology, School of Life Sciences, Northumbria University, Newcastle upon Tyne NE1 8STl, UK

## Abstract

A copious amount of scientific scrutiny has been dedicated to documenting typical and atypical human ageing, with a substantial body of work focusing upon the impact of lifestyle choices. One such lifestyle choice is that of diet and, in particular, micronutrient ingestion. Epidemiological studies have reported positive associations between B vitamin status and cognitive function, including negative associations between biological markers (i.e., homocysteine) of dysregulated one-carbon metabolism and cognitive function. This has led to a surge of randomised control trials (RCTs) investigations into B vitamin therapy. However, results have continuingly failed to show beneficial behavioural effects. Despite this, results reliably show treatment-related increases in B vitamin level and decreases in homocysteine level—both of which have been identified as risk factors for atypical ageing. In this paper we argue that it would be premature to conclude that B vitamin therapy has no potential and that more research is needed to systematically investigate the optimal dose, the therapeutic “window,” and individual differences in therapy responders and nonresponders. We start with a brief look at one-carbon metabolism and then consider the evidence from epidemiological studies and RCTs in relation to three specific B vitamins: folic acid (B9), pyridoxine (B6), and cobamides (B12).

## 1. Mechanism of Action

In 1992, two papers suggested that elevated levels of homocysteine may be a biological marker of abnormal one-carbon metabolism and that this may play a role in the aetiology of Alzheimer's disease (AD) [1, 2]. Behavioural support for this hypothesis comes from epidemiological work in healthy adults and patient populations, which demonstrates a negative association between homocysteine levels and cognitive function. In addition, it has been reported that elevated homocysteine levels are an independent risk factor for AD (see [3]).

One-carbon metabolism refers to the generation of one-carbon units, normally from serine, through association with a folic acid derivative; tetrahydrofolate (THF), to form 5, 10-methylenetetrahydrofolate and then 5-methyltetrahydrofolate. This, in turn, is used to methylate homocysteine, in a reaction catalysed by B12, and is used in the synthesis of methionine. Smith [3] outlines twelve biologically plausible mechanisms to explain the association between B vitamins, homocysteine, and dementia. However, Selhub et al. [4] state that the evidence for such mechanisms relies on animal and cell culture models and that there is “no supportive evidence to suggest the concentrations of homocysteine in brain or CSF reach levels considered neurotoxic, based on *in vitro* data” ([5]; page 115). 

## 2. Epidemiological Evidence

The relationship between B vitamin status and cognitive function gained momentum when reports began reporting associations between B vitamin status and cognitive function in the elderly. Such evidence is derived primarily from epidemiological studies. 

### 2.1. Healthy Elderly

Goodwin et al. [6] were the first to link nutritional status (using food record diaries and blood levels) and cognitive function in the healthy elderly population (mean age 71 years; *n* = 260). Essentially, Goodwin and colleagues used three different analyses to investigate this link: first, simple correlations were made between nutrient blood levels (protein, ascorbate, thiamine, riboflavin, pyridoxine, B12, niacin, and folate) and cognitive function (Russell revision of the Wechsler Memory test; Halstead-Reitan Categories Test). Using blood levels, the authors reported significant correlations between nutritional status and cognitive function for two nutrients (riboflavin B2 and ascorbate vitamin C) which could account for 2-3% of the variance in cognitive function. Using the participant's nutritional status they then extracted the bottom 5% and 10% of the sample and compared their blood nutrient levels to those of the rest of the sample (i.e., 90–95% of the participants). Based on variance in levels of ascorbate and B12 vitamins, there was a significant group difference on the Russell revision of the Wechsler Memory test. Following this first identification of a possible link between these nutritional factors and cognitive function, subsequent associations have been reported regarding the involvement of vitamin B in age-related cognitive decline (for a review see [7]).

One issue that has hindered the evaluation of the link between vitamin B intake and cognitive function is the fact that low B vitamin levels and high homocysteine (Hcy) levels are generally concomitant. Further research has attempted to investigate the relative importance of each of the B vitamins and Hcy for successful cognitive function. Riggs et al. [8] investigated the relationship between Hcy, B6, B9, and B12 with cognitive function in 70 males (mean age 66 years) as part of a normative aging study. The findings of this study revealed that lower concentrations of B9 and B12 and higher concentration of Hcy were associated with reduced spatial copying skills. Importantly, however, Hcy provided the strongest predictor of performance. In addition to this, results revealed higher concentrations of B6 to be related to better memory performance, leading the authors to suggest that the different B vitamins and Hcy may have differential effects upon different cognitive processes. An interesting observation from this study was that these associations were observed despite few participants having clinically relevant low vitamin B12 (<200 ng/L) and B9 (<3 *μ*g/L) levels. 

In similar research, Tucker et al. [9] used data from the Veterans Affairs Normative Ageing Study (NAS) to investigate associations between Hcy, B6, B9, and B12 with cognitive decline in 321 men (mean age 67 yrs; mean Mini-Mental State Exam—MMSE 27.2) over a 3-year period. In line with the results of Riggs et al. [8], Tucker and colleagues reported that higher concentrations of B9 (folate) and B12 and lower concentrations of Hcy were associated with better spatial copying skills. They also note B9 as being particularly relevant with regard to cognitive decline since an association was also found with the cognitive measure of verbal fluency. However, when considering the relative importance of the different vitamins on different components of cognition, Tucker et al. [9] reported vitamin B9 to be the only predictor of performance once the contribution of other vitamins was controlled for through covariance. In extending their research, Tucker and colleagues reported that only when vitamin B9 levels drop to <20 nmol/L is there a significant loss of spatial copying ability. Taken together this study demonstrated that folate may be particularly useful in protecting against cognitive decline, measured by spatial copying and language fluency tasks.

The differential impact of B vitamins on components of cognition has further been emphasised by Feng et al. [10], who investigated the relative associations between cognitive function and vitamins B9 and B12 and Hcy levels in 451 high-functioning elderly (>55 yr old) Chinese adult volunteers from the Singapore Longitudinal Ageing Study (MMSE ≥ 24). In this study, higher levels of Hcy were associated with poorer performance on the Block Design task (a measure of visuospatial motor skill) and the Symbol Digit Modality Test (a measure of executive functioning). Further, higher levels of vitamin B9 were associated with better performance of the Rey Auditory Verbal Learning Test and verbal fluency, and, finally, levels of vitamin B12 were not associated with performance on any of the cognitive tasks. 

It is worth mentioning one study that investigated these associations across a broader age group, rather than restricting the sample to older adults. Using a sample of 2,871 participants from the Northern Manhattan Study, Wright et al. [11] reported an association between higher Hcy levels and lower mean MMSE scores for adults older than 65 years but not for adults between the ages of 40 and 64 years. Adjusting for B12 deficiency and sociodemographic factors, the mean MMSE was 2.2 points lower for each unit of increase in the log Hcy level.

On the basis of the studies summarised in this section, it is apparent that clear associations have been established between B vitamin levels, as well as Hcy levels, and cognitive function in older healthy adults. There are also suggestions of independent and differential effects of some B vitamins on subcomponents of cognitive processing; however, it is clear that further research is needed to fully understand these associations with regard to process-specific effects and “critical” levels. 

The next section will consider evidence of a relationship between B vitamin status and the risk of developing Mild Cognitive Impairment (MCI) and Dementia.

## 3. MCI and Dementia

The chance of developing MCI and/or dementia in later life is exacerbated by a number of risk factors. Although the biggest risk is increased age, there is substantial research investigating risk factors associated with lifestyle choices, one of these being nutritional deficiencies. Droller and Dossett [12] first reported a link between low vitamin B12 level and dementia that was independent of pernicious anaemia (B12 deficiency anaemia). Subsequent to this, other studies have attempted to establish the relative importance of each B vitamin and Hcy as risk factors for developing MCI and dementia. For example, Quadri et al. [13] examined associations between plasma Hcy, B9, and B12 and the probability of developing MCI, dementia of the Alzheimer's type (AD), or vascular dementia (VaD). Quadri and colleagues categorised 228 participants into 3 groups: an elderly control group (*n* = 55, mean age 76 years); those diagnosed with MCI (*n* = 81, mean age 76 years); and those diagnosed with dementia (*n* = 92, mean age 80 years). Results revealed that vitamin B9 concentrations were lower in the dementia groups and MCI group compared to those of the control group. Hcy was also higher in the dementia groups in comparison to that of the elderly control group, and the proportion of people in the dementia groups with higher than normal Hcy level (<14.6 *μ*mol/L) was greater, in comparison to that of the control group. The sample was further subcategorised using tertiary split, and odds ratios were calculated. Results suggest that those with the highest Hcy levels (>14.6 *μ*mol/L) and the lowest B9 levels (<13.5 nmol/L) were more than 3 times more likely to develop AD. This is in line with Tucker et al. [9], who also report “critical” values of B9 levels below which cognitive impairment is typically observed, albeit in a different value and in a nondemented population.

In further research, Ramos et al. [14] utilised the Sacramento Area Latino Study on Aging (SALSA) to investigate the relationship between vitamin B9 status, cognitive function, and dementia diagnosis in 1789 community-dwelling individuals of Latino ethnic background over 60 years of age. Employing a 4-stage regression analysis, results revealed that B9 status was correlated with performance on all 7 cognitive tasks (model 1) and was correlated with 6 cognitive tasks after Hcy (model 2), and vitamin B12 and creatinine (model 3) were controlled for statistically. However, when demographic variables and depression scores were added (model 4), vitamin B9 only correlated with 3MSE (a measure of global cognitive ability) and delayed memory recall. Secondary analyses demonstrated that the odds ratios (OR) (controlling for Hcy, B12, creatinine, demographic variables, and depression score) for (i) low 3MSE score (<78) and (ii) dementia diagnosis decreased with increasing B9 concentrations. The OR for Hcy did not remain significant after controlling for vitamin B9. In addition, Haan et al. [15] investigated the relationship between Hcy, vitamin B9, and vitamin B12 and the risk of developing dementia or cognitive impairment without dementia (CIND) in 1405 participants aged 60–101 years old. In contrast to Ramos et al. [14], Hann et al. reported that it is Hcy (not B9) that is associated with a greater risk of dementia or CIND and that higher B12 concentrations may reduce this risk. To further complicate matters, Mooijaart et al. [16] also investigated the relationship between (i) Hcy, (ii) B12, and (iii) B9 and cognitive decline. Five hundred and ninety-nine elderly participants completed a test battery at 85 years of age and yearly thereafter until they were 89 years of age. Hcy, vitamin B12, and B9 were measured at the first (i.e., 85 years) and the last (i.e., 89 years) testing sessions. A cross-sectional analysis at 85 years showed a negative relationship between Hcy and MMSE, a positive association between B9 and cognitive function, but no significant relationship between B12 and cognitive function. A prospective analysis showed that concentrations of Hcy, vitamin B12, and B9 at 85 years were not predictive of cognitive decline over the study period. However, it should be noted that participants were not screened for health or demographic variables (shown to be important by Ramos et al. [14]). It is difficult to reconcile such opposing results. However, some of the conflicting results may be due to different methods and the populations being studied.

One potentially important methodological confound between studies in this area is the method used to measure vitamin B status. This potential conflict is highlighted by the results of Clarke et al. [17] who investigated the associations between cognitive decline and levels of vitamins B12, B9, and Hcy (measuring holoTC, tHcy, MMA, and vitamins B12 and B9 levels). Cognitive function was assessed in 1648 participants at baseline (mean age 75 years; mean MMSE 26.2) and again at a minimum of 3 time points over a 10-year period. Six hundred and ninety-one participants survived until the 10-year followup (mean age = 72 years; mean MMSE = 27.3). Cross-sectional analyses at baseline and at the 10-year followup revealed significant associations between several factors, for example, between tHcy and cognitive function, between holoTC (a marker of reduced vitamin B12 status) and cognitive function, and between MMA and cognitive function. Longitudinal analyses revealed that a doubling in holoTC concentrations (50–100 pmol/L) was associated with a 30% slower rate of cognitive decline. Conversely, a doubling in tHcy (from 10 to 20 *μ*mol/L) or MMA (0.25 *μ*mol/L) was associated with >50% more rapid cognitive decline. Interestingly, in contrast to the previous studies, Clarke and colleagues found no association between cognitive function, total vitamins B12 or B9 levels. The authors suggest these may be less accurate markers of B vitamin status in comparison to holoTC and MMA. An additional consideration is that there is evidence to suggest that the relationship between vitamin B12 status and cognitive function in older individuals may be moderated by APOE (Apolipoprotein E). Feng et al. [18] investigated this possibility. Five hundred and thirty-nine adults (mean age 65 years) completed a number of cognitive function tests including the MMSE. The MMSE was completed at baseline (all participants had had an MMSE >21) and ~18 months and ~38 months thereafter. Results suggest that the relationship between B12 and MMSE score was stronger in the carriers of the APOE *ε*4 (the dysfunctional isoform) than noncarriers, suggesting that APOE *ε*4 moderates this relationship. However, it should be noted that this moderating effect was not present when the relationship was analysed in a subsample of 416 “cognitively normal” (i.e., nondemented) adults.

It is clear from extant findings that Hcy levels and vitamin B status are related to MCI and dementia in the elderly. However, to date the nature of the relative contribution of each of the B vitamins and Hcy is not fully understood. In addition to this, there is evidence of “critical” levels and individual differences in vulnerability that deserve further research. 

As a result of these associations, a substantial research has now been conducted to ascertain whether B vitamin supplementation can slow age-related cognitive decline. The next section will discuss some of the empirical studies that have addressed this research question.

## 4. Vitamin B Supplementation Studies

There has been substantial research conducted to date which has investigated whether B vitamin supplementation can aid cognitive function in the elderly. The majority of the evidence obtained using experimental methodology has provided no evidence of efficacy of vitamin B supplementation. However, it is clear that more research is needed to answer a number of fundamental questions related to any treatment: (1) what is the optimal dose required; (2) what is the optimal treatment period; (3) is there a therapeutic window; and (4) what are the individual differences in responders and nonresponders. To date, these questions have not been sufficiently investigated or satisfactorily answered. In addition to this, research needs to further explore the interaction between approved medical treatments for dementia and nutritional supplements whilst controlling for important extraneous variables (e.g., genetic and demographic variables). 

One of the main problems with research in this domain has been methodological. For example, Yukawa et al. [19] assessed the effects of 60-day treatment with vitamin B9 (15 mg per day) in 36 (mean age 56 years old) vitamin B9 deficient volunteers (defined as <4 ng/mL serum folate), all with neurological disease. Results suggested that B9 administration improved neurological symptoms in 24 of the 36 cases after 2 months (8 of these individuals have been diagnosed with dementia). However, Yukawa et al. did not include a placebo control group in their study. Similarly, Nilsson et al. [20] investigated the effects of an oral supplementation of vitamins B12 (1 mg day) and B9 (5 mg/day) for 2 months. Thirty-three patients (mean age 78 years) were classified with mild, moderate, or severe dementia (categorized according to DSM III-R criteria). The patients with severe dementia were too ill to participate and were excluded from this study. The remaining 28 patients were subcategorised into two groups based on their Hcy levels (those having a plasma Hcy level <19.9 *μ*mol/L and those above). Results revealed that the patients with high Hcy (>19.9 *μ*mol/L) showed clinical improvements after oral supplementation of vitamin B12. However, once again the study failed to implement a placebo control group.

In studies that have used more rigorous methods, the results are much clearer. The conclusions obtained from these scientific investigations indicate that vitamin B supplementation has no effect upon cognitive function alone or in combination (for reviews, see [21–23]). However, it should be noted that individual studies have varied substantially with regard to treatment, treatment dose, duration of treatment regime, study population, and method of assessment of cognitive function. In addition, some studies have used a very small sample size, which inevitably increases the probability of reporting findings that support a null hypothesis, due to decreased power. Some of the relevant problems will be highlighted in the remainder of this paper, where we will consider the evidence of monotherapy and multivitamin therapy. 

## 5. Monotherapy with Vitamins B6, B9, and B12

The majority of monotherapy studies have elected to study vitamin B9 and have kept the experimental design simple (something that we would advocate) by comparing one B vitamin with placebo. However, one study has investigated the effect of B6, B9, and B12 against a placebo. Bryan et al. [24] utilised a placebo-controlled design to investigate the effect of 35 days of B vitamin supplementation on cognitive function and mood in three age ranges: (1) young adults (20–30 years old, *n* = 56), (2) middle-aged adults (45–55 years old, *n* = 80), and (3) older adults (65–92 years old, *n* = 75). Participants were randomly allocated to one of 4 treatment conditions (1) B9 (0.75 mg), (2) B12 (0.015 mg), (3) B6 (75 mg), or (4) placebo. Results revealed no significant evidence of a treatment effect on cognitive function and no significant effect on subjective mood. 

## 6. Vitamin B6

Few studies have investigated the therapeutic value of vitamin B6 as a monotherapy. Deijen et al. [25] utilised a placebo-controlled trial investigating the effects of 12 weeks of daily ingestion of vitamin B6 (20 mg). Seventy-six healthy volunteers (mean age 73 years) were assigned to treatment or placebo groups using a matched pairs methodology. Twelve participants in the placebo condition and 4 participants in the treatment condition were defined as marginally B6 deficient at baseline (PLP <20 nmol/L or *α*-EAST >1.98). However, these participants were retained in the study, thereby raising some doubts about the match pairing methods used (participants were reportedly matched on age, B6 status, and IQ). Results provided minimal support for a treatment effect, using multiple bivariate correlations to examine changes in cognitive performance and PLP baseline levels. Although the authors did report some significant effects, they failed to control for multiple comparisons and did not report the associations obtained in the placebo group.

## 7. Vitamin B9

Numerous studies have investigated the therapeutic value of vitamin B9 folate as a monotherapy. However, the disparity in methods makes it almost impossible to form a rational conclusion, other than one that requests more research to be carried out. Pathansali et al. [26] utilised a placebo-controlled design to investigate the effect of daily ingestion of vitamin B9 (5 mg) over a 4-week period on cognitive function in 24 healthy older adults (mean age 73 years; MMSE >27) with normal baseline B9 levels (6.3 ± 2.4 *μ*g/L). Results demonstrated no effect of vitamin B9 on psychomotor function. B9 levels increased following treatment, and Hcy levels were reduced. Over a longer treatment period and implementing a larger dose, Sommer et al. [27] utilised a placebo-controlled design to investigate the effects of daily vitamin B9 treatment (10 mg) over a 10-week supplementation period in 7 patients (mean age 77 years old) suffering from dementia (classified by DSM-III-R) who also presented low vitamin B9 levels (defined as serum B9 between 2 and 5 mcg/L or RBC B9 between 127 and 452 mcg/L and B12 above 200 ng/L). Results show no effect of B9 supplementation in comparison with placebo. Connelly et al. [28] investigated a longer treatment period but reduced the daily dose. In a placebo-controlled trial, these researchers investigated the effect of vitamin B9 treatment on 41 (mean 76 years; mean MMSE 23.49) participants with probable AD (meeting the NINCDS-ADRDA diagnostic criteria). Participants were allocated to daily treatment (1 mg of B9) or placebo for 6 months. At the same time, all participants started cholinesterase inhibition (ChI) treatment. However, the type of ChI and dose varied across participants, depending on their clinical response. Results show that 23 out of 28 participants in the folic acid groups and 7 out of 18 participants in the placebo group were classified as NICE (National Institute for Health and Clinical Excellence) responders (defined as having good response after 6 months treatment, according to NICE criteria; NICE 2001). Within group change from baseline showed improvements following B9 on activities of daily living and social behaviours but no change in MMSE score. However, it is very difficult to delineate the effects of ChI in this study (and possible interaction with B9 supplementation), given the personalised medical treatment. 

In a large study, Durga et al. [29] implemented a placebo-controlled, between-subjects design to investigate the effects of daily B9 supplementation (0.8 mg of B9) for 3 years on cognitive function in 818 individuals (mean age 60 years: MMSE at baseline >24). Four hundred and four participants were allocated to the intervention group. Within group comparisons of change data revealed better cognitive performance following B9 treatment. However, as with Connelly et al. [28], within group comparisons were not supported by between group differences, and the results should therefore be treated with some caution. 

## 8. Vitamin B12

In a very small study, Seal et al. [30] utilised a placebo-controlled design to investigate the effects of two doses of vitamin B12 (0.01 mg or 0.05 mg) ingested daily for 1 month by 31 older adults (mean 81.4 years; mean MMSE 18.23) with subnormal vitamin B12 levels (serum B12 between 100–150 pmol/L). Results revealed that 0.05 mg B12 daily increased serum B12 but did not affect cognitive function. In a larger study conducted over a longer period of time and using a larger dose, Hvas et al. [31] utilised a placebo-controlled trial to investigate the effect of 3-month treatment with vitamin B12 (1 mg intravenous). One-hundred and forty participants (mean age 74.5 years; mean MMSE = 26.5) were randomly assigned to either treatment or placebo groups. Participants had increased P-MMA (0.4–2 *μ*mol/L) and high tHcy (mean: 13 *μ*mol/L) at baseline. Results revealed no significant effect of treatment on cognitive performance. 

## 9. Multi-B-Vitamin Therapy

A number of studies have investigated the effect of B vitamin treatment in combination with two or more B vitamins. Stott et al. [32] implemented a placebo-controlled trial to investigate the effect of a 12-week vitamin supplementation programme on Hcy levels (assessed at baseline and 3 months later) and cognitive function (assessed at baseline and 6 and 12 months after randomisation) in 185 elderly (>65 years old) patients with vascular disease. Patients were randomly allocated to a placebo condition or one of seven different treatment conditions. Treatments were (1) B9 (2.5 mg) plus vitamin B12 (0.5 mg), (2) vitamin B6 (25 mg), and (3) riboflavin B2 (25 mg). Seven treatment comparisons were undertaken, comprising the three treatment conditions alone (i.e., 1, 2, and 3) and in combination with each other (i.e., 1+2, 1+3, 2+3, and 1+2+3). The results suggested that B9 with B12 could decrease Hcy level but had no effect on cognitive function.

Other studies have combined intake of B vitamins with other vitamins that are not from the B vitamin group. In a large study, over a long treatment period, Clarke et al. [33] utilised a placebo-controlled trial to investigate the effects of aspirin (81 mg), vitamin B (B9 at 2 mg; B12 at 1 mg), and vitamins C and E (200 mg and 500 mg) ingested daily for 12 weeks on cognitive function (measured by MMSE and the cognitive part of the Alzheimer's Disease Assessment Scale, ADAS-Cog). One hundred and forty-nine participants (median age = 75 years, median MMSE = 21, and median Hcy = 12.4 *μ*mol/L^−1^) were randomly allocated to treatment and placebo arms (6 different groups), with 142 of these individuals returning for followup. At baseline (*N* = 149), data were split by age (<75 and >75 years old) and severity of cognitive impairment (interquartile split). Results revealed a negative correlation between cognitive function and Hcy levels and a positive association between B9 and cognitive function but no association between B12 and cognitive function (these associations remained after adjustment for age). After 12 weeks supplementation with vitamin B (B9 at 2 mg, B12 at 1 mg), results revealed significant increases in B9 and B12 and significant decreases in Hcy. However, there was no effect on cognitive function. Lewerin et al. [34] utilised a placebo-controlled trial to investigate the effect of 4 months of treatment (0.5 mg B12, 0.8 mg B9 acid, and 3 mg B6) on nine cognitive tasks (digit span forward and backward, identical forms, visual reproduction, synonyms, block design, digit symbol, Thurstone's picture memory, and inductive reasoning task) and five indices of movement (movement time, postural phase, locomotor phase, manual phase, and simultaneity index) in 209 community-dwelling elderly volunteers (mean age 75.41 years, mean tHcy = 16.95 *μ*mol/L, and MMSE was not completed). At baseline, tHcy and MMA correlated with cognitive function and movement; however, B9 and B12 levels did not. Four months of treatment had no effect on cognitive function, but did decrease tHcy and MMA levels.

Eussen et al. [35] implemented a placebo-controlled, between-subjects design to investigate the effect of a 24 week dietary supplementation period on cognitive function in elderly (>70 years old) participants with mild vitamin B12 deficiency (defined as B12 concentration between 100 and 200 pmol/L, or between 200 and 300 pmol/L with concomitant high MMA levels and low creatinine concentrations). Treatments consisted of (1) B12 (1 mg daily, *n* = 54), (2) B12 + B9 (1 mg + 0.4 mg *n* = 51), and (3) placebo (*n* = 57). Results demonstrated no effect of either treatment on cognitive function.

Over a longer treatment period and using larger doses, Aisen et al. [36] utilised a placebo-controlled-between-subjects design to investigate the effect of B vitamin supplementation (5 mg B9, 25 mg B6, and 1 mg B12 daily) for 18 months on cognitive decline in those with mild to moderate AD (mean age 76 years with MMSE between 14 and 26). Two hundred and two participants received the active treatment and 138 received the placebo. Results revealed no beneficial effect of treatment. Similar conclusions were made by McMahon et al. [37] who investigated the effects of a 2-year daily treatment regimen (1 mg of B9, 0.5 mg of B12, and 10 mg of B6) on Hcy and verbal and nonverbal cognitive function in participants aged 65 years and older with a baseline Hcy level of at least 13 *μ*mol/L. Results revealed reduction in Hcy following treatment at 6, 12, 18, and 24 months after randomisation; however, there was no effect on cognitive function. Smith et al. [38] implemented another study over a 24-month period to investigate whether 24-month supplementation with high doses of B vitamins (B9 0.8 mg/d, B12 0.5 mg/d, and B6 20 mg/d) resulted in slowing the rate of cerebral atrophy in elderly (>70 years old) participants with MCI in comparison to placebo. Results revealed that treatment preceded reduction in tHcy and a 30% reduction in the rate of atrophy, which increased to 53% reduction in those who had shown the greatest baseline levels of tHcy (>13 *μ*mol/L). There was no effect of treatment on those with the lowest baseline levels of tHcy (<9.5 *μ*mol/L). The authors do not report any data relating to cognitive performance. In one of the longest treatment studies, Kang et al. [39] utilised a placebo-controlled design to investigate the effects of B vitamin supplementation (comprising 2.5 mg B9, 1 mg of B12, and 50 mg of B6) in two thousand and nine elderly (mean age 72 years) women with cardiovascular disease and CVD risk factors (1002 allocated to treatment group) over a five-and-half-year period using a telephone cognitive battery measuring (1) general cognition (Telephone Interview of Cognitive Status (TICS)), (2) verbal memory (delayed recall of the TICS 10-word list and the immediate and delayed recalls of the East Boston Memory Test), and (3) category fluency (asked to name as many animals as possible in 1 minute). Results revealed no effect of treatment.

## 10. Conclusions

As summarised in this paper, there appears to be some robust epidemiological evidence linking B vitamin status and Hcy with cognitive decline; however, there is still debate about which marker is the best predictor, and there are a number of unresolved issues around vulnerability. Despite this promising link, the empirical evidence that has been put forward in the literature relating to the potential of vitamin therapy has proven very disappointing. However, once again there are fundamental questions that need to be considered further. It is clear from these and other findings reported in this paper (together with inconsistencies that have been highlighted) that further research is warranted in this domain.
